# Research on the factors affecting the adoption of health short videos by the college students in China: unification based on TAM and UTAUT model

**DOI:** 10.3389/fpsyg.2025.1547402

**Published:** 2025-07-08

**Authors:** Pu Han, Senling Liu, Deqiang Zhang, Xiong Li, Xiaoyan Li

**Affiliations:** ^1^School of Management, Nanjing University of Posts and Telecommunications, Nanjing, China; ^2^Jiangsu Provincial Key Laboratory of Data Engineering and Knowledge Service, Nanjing, China; ^3^School of Basic Medical Sciences, Nanjing Medical University, Nanjing, China

**Keywords:** health short videos, health information dissemination, information adoption behavior, Chinese college students, integrated model

## Abstract

**Background:**

Health short videos, as an emerging mode of information dissemination, play a crucial role in enhancing health awareness and promoting healthy behavior among college students. It is crucial to optimize both the content and dissemination strategies of these videos to amplify their impact on health communication and to enhance the health literacy of this demographic.

**Objective:**

This study aims to construct a comprehensive model to explore the key factors influencing the use of health short videos among Chinese college students, including perceived usefulness, perceived ease of use, and performance expectations, among other factors. The goal is to supply theoretical foundations and practical guidance for optimizing health short video content and dissemination strategies, thereby enhancing college students’ health literacy and quality of life.

**Methods:**

An online survey was conducted among Chinese college students to investigate their inclination to acquire health information from health short videos. Based on TAM and UTAUT frameworks, a model was constructed to examine the factors influencing college students’ adoption of health information from health short videos. Through structural equation modeling, the study analyzed the impact of health short videos on the information adoption behavior of college students.

**Results:**

This study included a total of 296 Chinese college students. Results from the structural equation model indicated that perceived usefulness (*β* = 0.443, *p* < 0.001), perceived ease of use (*β* = 0.398, *p* < 0.001), performance expectancy (*β* = 0.434, *p* < 0.001), effort expectancy (*β* = 0.456, *p* < 0.001), social influence (*β* = 0.443, *p* < 0.001), information quality (*β* = 0.427, *p* < 0.001), and perceived trust (*β* = 0.482, *p* < 0.001) significantly positively influenced intention to adopt health short videos. Conversely, perceived risk (*β* = −0.415, *p* < 0.001) and perceived disease threat (*β* = −0.480, *p* < 0.001) had significant negative effects on adoption intention. Additionally, facilitating conditions (*β* = 0.421, *p* < 0.001) positively influenced adoption behavior, and adoption intention significantly affected adoption behavior (*β* = 0.406, *p* < 0.001).

**Conclusion:**

The adoption of health short videos by Chinese college students is primarily positively influenced by factors such as perceived usefulness, ease of use, performance expectancy, effort expectancy, social influence, information quality, and trust. Conversely, perceived risk and disease threat negatively affect their usage. Therefore, to promote college students’ continuous attention to and adoption of health short videos, the quality and credibility of health short videos should be improved, the user interface design should be optimized, usability should be enhanced, and social influence strategies should be used to enhance the attractiveness and persuasiveness of health information.

## 1 Introduction

Health serves as the cornerstone of personal well-being and quality of life, fundamentally underpinning societal stability and development. A healthy workforce drives enhanced productivity and creativity, fundamentally underpinning individual well-being, family and societal stability, and economic progress (Hoy-Ellis, 2023). The “Healthy China 2030” initiative highlights that public health serves as a crucial barometer of national prosperity and individual well-being (Xuan et al., 2024). Under the strategic background of “Healthy China 2030,” “healthy living” has emerged as a universal pursuit, with individuals increasingly seeking daily access to health information to elevate their overall well-being. The 14th Five-Year National Health Plan advocates for establishing comprehensive media channels to deliver health education and promotion, calling on healthcare institutions and professionals to actively engage in health education initiatives. The efficient dissemination of health information is crucial for improving national health standards (Liu et al., 2020).

Initially, American scholar Rogers defined health communication as the process of translating medical research findings into public health knowledge to enhance the quality of life and health, with a primary focus on preventing diseases such as HIV/AIDS, early-stage cancers, and substance abuse (Rogers, 1994). Subsequently, Rogers expanded this definition to include interpersonal communication on all health-related topics, a perspective that gained widespread scholarly acceptance (Rogers, 1996). With the rapid advancement of internet technology, researchers have explored its role in online health information dissemination (Tsao et al., 2021; Schillinger et al., 2020) noting that offline activities effectively complement this process (Gilmore et al., 2020). With the rise of major short video platforms like “Miaopai” and “Wesee” in 2013, short videos have emerged as the primary way for people to access information (Shi et al., 2023). Currently, short video content has transcended entertainment to incorporate professional knowledge dissemination and health education (Gao et al., 2021), serving as a bridge for interaction between individuals and health information. Health short videos are defined as videos where professional doctors or institutions condense health knowledge and information through new media platform editing and processing, with a duration of less than 5 min. These videos cover all aspects of daily life, aiming to improve public quality of life and health (Song et al., 2021). According to relevant data analysis (Xie et al., 2024), health short videos on platforms like “Little Red Book” and “Douyin” have achieved cumulative views exceeding 100 billion within just a few years, emerging as a pivotal channel for public health information acquisition.

While health short videos on major platforms are widely accessible and diverse, their quality remains inconsistent, often lacking scientific rigor and accuracy. This inconsistency leads to divergent dissemination effects and audience reception, characterized by a pronounced “head effect,” where a minority of creators receive disproportionate attention. The majority of creators, however, experience low dissemination efficiency (Osman et al., 2022). Although existing research has explored factors influencing the adoption of health short videos (Huo and Huo, 2024; Wang et al., 2024), few studies have conducted specific and independent analyses of college students’ adoption behavior. College students, as key information recipients and disseminators in society, are at a critical stage of value and lifestyle formation, facing numerous challenges in making healthy choices (Deliens et al., 2015). As a key audience for health communication, college students demonstrate a higher receptivity to health information, a trait that is vital for facilitating the widespread dissemination and popularization of health knowledge. Understanding how they process health short video information can enhance college students’ health awareness and decision-making capabilities, while also enabling them to better comprehend their own health status. Health institutions can leverage this understanding to optimize promotion strategies, ensuring that college students acquire accurate health knowledge and enhance their health literacy (Woolf et al., 2005). Hospitals can also tailor health promotion content by identifying the specific health needs and behavior of college students, thereby fostering healthier behavioral patterns.

The concept of “information adoption behavior” was initially proposed by Stephani and Wendy (Sussman and Siegal, 2003), who defined it as the process whereby individuals select new information based on their attitudes and beliefs. Subsequently, Cheung et al. (2008) further elaborated on information adoption behavior as the process of leveraging information to achieve specific goals and actively seeking behavioral guidance. With the advent of the Technology Acceptance Model (TAM) (Davis, 1989) and the Unified Theory of Acceptance and Use of Technology (UTAUT) (Ajzen and Madden, 1986), these theoretical models have garnered extensive scholarly recognition in the domain of health information behavior research. Studies have demonstrated that the TAM model (Venkatesh et al., 2003; Ayeh, 2015) and the UTAUT model (Mäntymäki and Salo, 2013; Philippi et al., 2021) are effective in analysing factors affecting user information adoption behavior. However, research that simultaneously applies both TAM and UTAUT models to study information adoption behavior among college students remains relatively scarce. Current research frequently employs models such as the Information Adoption Model (Zhou, 2022; Tao et al., 2020), the Theory of Planned Behavior (Yang and Wu, 2021), and the Dual Process Theory (Zhao et al., 2022) to explore the mechanisms and influencing factors of health information adoption. The TAM and its derivative, the UTAUT model, provide a comprehensive framework for explaining the factors influencing college students’ adoption of health information, offering deeper insights into the process of their behavior. Therefore, by focusing on the novel research topics of health short videos and college students, this study aims to integrate the Technology Acceptance Model (TAM) and the Unified Theory of Acceptance and Use of Technology (UTAUT) to explore how factors such as information quality, perceived usefulness, and perceived ease of use in health short video content and format influence college students’ information adoption behavior. The findings will assist college students in effectively absorbing health information from short videos, cultivating healthier lifestyles, and enhancing the quality and dissemination of health information. This, in turn, will enhance the effectiveness of health knowledge dissemination, boost user engagement, and ultimately improve the sustainability of health information communication among college students (Abdelhamid, 2018).

The structure of this study is organized as follows: Section 2 introduces the TAM and UTAUT models and formulates hypotheses based on these frameworks. Section 3 details the sample selection, data collection, and data analysis methods. Section 4 presents the findings from reliability and validity analyses, variance analyses, and structural equation model. Section 5 interprets these findings. Finally, Section 6 and Section 7 address the research implications and limitations.

## 2 Literature review and research hypotheses

### 2.1 Tam

The Technology Acceptance Model (TAM), first proposed by Davis (1989), is rooted in the Theory of Planned Behavior and the Theory of Reasoned Action, aiming to investigate the mechanisms through which individuals adopt and accept new information technologies. The TAM model posits that the user’s behavioral intentions determine their adoption behavior, where behavioral intention is shaped by the attitude toward use and perceived usefulness, while external factors determine perceived usefulness and perceived ease of use (Ajzen and Madden, 1986). Therefore, TAM has been widely applied across various industries to analyse user information behavior. For instance, in the field of artificial intelligence (AI) (Park, 2020; Sagnier et al., 2020; Shao et al., 2025), studies leveraging TAM have demonstrated that perceived ease of use and perceived usefulness significantly impact technology acceptance (Mohr and Kühl, 2021). In online education (Al-Adwan et al., 2023; Li, 2023), studies have indicated that these factors positively influence college students’ attitudes, behavioral intentions, and actual usage of AI systems (Tao et al., 2022). In business and service sectors (Ruiz-Herrera et al., 2023; Agag and El-Masry, 2016), TAM highlights that attitude toward use, perceived usefulness, ease of use, and perceived trust are crucial for influencing consumer behavior. Similarly, in telemedicine (Kamal et al., 2020; Lu et al., 2024), TAM identifies perceived ease of use, perceived trust, and perceived risk as key factors affecting people’s willingness to use these services. In short video studies (Guo et al., 2025; Wang et al., 2022), it has been found that perceived usefulness and perceived ease of use have a significant impact on users’ attitudes and behavioral intentions toward using short videos. Existing research primarily uses TAM to assess the intrinsic characteristics of information technologies, their usefulness, and ease of use, thereby analyzing factors influencing user acceptance and information behavior. However, these factors may be too broadly generalized, potentially overlooking individual differences and situational contexts (Chen et al., 2023). Thus, other factors may need to be incorporated to complement the model.

### 2.2 UTAUT

Venkatesh et al. (2003) proposed the Unified Theory of Acceptance and Use of Technology (UTAUT), building on the TAM model. UTAUT integrates four core variables: performance expectancy, effort expectancy, social influence, and facilitating conditions. It also considers moderating factors such as gender and age to enhance its predictive capability (Ajzen and Madden, 1986). Due to its comprehensiveness and applicability (Al-Saedi et al., 2020), UTAUT has been widely adopted to study user acceptance behavior toward technology (Abbad, 2021; Raffaghelli et al., 2022). However, to increase its relevance in various contexts, additional factors are often introduced to address specific needs arising from different technological characteristics and cultural backgrounds (Zhao and Wang, 2020). For example, Dai et al. (2020) introduced the concepts of resistance to change (RC) and technostress anxiety (TA) to study caregivers’ acceptance of wearable devices for dementia patients. Huang (2023) incorporated the Theory of Planned Behavior (TPB) to supplement the UTAUT model in studying users’ intentions toward VR tourism behavior. Xu et al. (2023) explored the intention of users to seek travel information on short video platforms by incorporating various technological affordances and perceived enjoyment as supplements to the UTAUT model.

The TAM places greater emphasis on the intrinsic characteristics of technology and users’ direct experiences with it, whereas the UTAUT underscores the influence of social and environmental factors (Zhao and Wang, 2020). Although UTAUT extends TAM by incorporating additional constructs, the concepts of perceived usefulness and perceived ease of use from TAM offer a succinct and robust explanation for the initial adoption intention of health short videos. Integrating TAM and UTAUT addresses certain limitations inherent in UTAUT alone. By embedding the core elements of TAM into the UTAUT framework, the model’s comprehensiveness in explaining user technology acceptance is significantly enhanced. This integrated approach facilitates an analysis of the factors influencing technology acceptance from both the individual user perspective and the broader socio-technical environment (Rouidi et al., 2022; Al-Adwan et al., 2025).

Therefore, this study constructs a model to investigate the factors influencing college students’ adoption behavior of health short videos, based on the TAM and UTAUT models. The model introduces ten independent variables: information quality, perceived usefulness, perceived ease of use, facilitating conditions, perceived risk, perceived disease threat, effort expectancy, performance expectancy, social influence, and perceived trust. Adoption intention is proposed as a mediating variable, while adoption behavior serves as the dependent variable. This comprehensive model aims to explore the complete path of college students’ adoption behavior toward health short videos.

### 2.3 Research hypotheses

Perceived usefulness (PU) refers to users’ belief that using a specific system or technology will enhance their performance or effectiveness (Davis, 1989). Sussman et al. positioned perceived usefulness as a critical factor in the context of knowledge acceptance, demonstrating its mediating role between information and adoption (Sussman and Siegal, 2003). In this study, perceived usefulness is defined as college students’ perception that watching health short videos will improve their health. For college students, who often experience high academic stress and limited access to formal health education, health short videos serve as a convenient and visual means to acquire essential health knowledge. These videos offer practical guidance on nutrition, mental health, and physical activity that aligns with students’ daily needs, making them more likely to perceive the content as highly useful. This perceived usefulness, in turn, strengthens their trust in such platforms, establishing it as a primary driver of adoption intention. Therefore, we propose:

*H1*: Perceived usefulness significantly influences adoption intention.

Perceived ease of use (PEU) refers to individuals’ perception of how easy and effortless it is to use a specific technology or tool (Davis, 1989). College students, as digital natives, are well-acquainted with using mobile devices and social media platforms for both learning and entertainment. Short video platforms, characterized by their intuitive interfaces and minimal learning curves, enable students to quickly access health-related content even during brief intervals between classes or study sessions. Consequently, the perceived ease of use is anticipated to significantly influence their intention to adopt these platforms for health information. Based on this understanding, the following hypothesis is proposed:

*H2*: Perceived ease of use significantly influences adoption intention.

Performance expectancy (PE) refers to the degree to which individuals believe that using a new system will benefit them (Davis, 1989). College students may anticipate that watching health short videos will provide actionable insights for managing stress, improving sleep quality, or adopting healthier eating habits—all of which are directly relevant to their current lifestyle challenges. When students perceive that interacting with these videos enhances their well-being, their intention to continue using them is likely to increase. Based on these considerations, the following hypothesis is proposed:

*H3*: Performance expectancy positively and significantly influences adoption intention.

Effort expectancy (EE) is commonly understood as the amount of effort individuals believe they need to exert when using a new system (Davis, 1989). Given the hectic schedules of college students, they tend to favor tools that demand minimal effort to retrieve health information. The more effortlessly college students perceive the process of searching for health short videos to be, the stronger their intention to adopt this technology. Based on this, the following hypothesis is proposed:

*H4*: Effort expectancy positively and significantly influences adoption intention.

Social influence (SI) refers to the degree to which an individual’s behavior is influenced by significant others (Ajzen and Madden, 1986). For college students, peer groups exert a substantial influence on shaping attitudes toward new technologies. When peers frequently share or recommend health short videos, individuals are more inclined to perceive these resources as credible and valuable. Furthermore, endorsement from parents or instructors can also heighten students’ interest and willingness to engage with health-related content. Thus, social influence emerges as a critical determinant of adoption intention within this demographic. Based on this, we propose:

*H5*: Social influence positively influences adoption intention.

Information quality (IQ) refers to the persuasiveness of the presented information and its attributes, such as authenticity and completeness (Cheung et al., 2012). College students, as an educated demographic, attach great importance to the accuracy and scientific validity of health information. They are more inclined to adopt health short videos if the content is evidence-based, logically structured, and presented by credible professionals. Conversely, poor information quality, such as misinformation or oversimplification can erode trust and discourage sustained engagement. Based on these considerations, we propose:

*H6*: Information quality positively influences adoption intention.

Perceived trust (PT) refers to the level of confidence individuals have in a service, product, or platform, including trust in brands, individuals, or viewpoints (Lăzăroiu et al., 2020). Trust in the influencers or institutions behind health short videos plays a pivotal role in students’ decision-making processes. Many college students follow online health educators or medical professionals whose expertise they esteem. When students associate the content with trustworthy figures or reputable organizations, they are more inclined to accept the information and act upon it. Based on this understanding, this study proposes:

*H7*: Perceived trust positively influences adoption intention.

Perceived risk (PR) involves the uncertainty and potential negative consequences individuals associate with using a new system or product (Yi et al., 2020). While many health short videos provide valuable insights, some may contain misleading or unverified claims that pose risks to users. College students, who may lack the skills to critically evaluate health content, might hesitate to adopt such videos due to concerns about inaccurate advice or harmful recommendations. The perception of these risks can significantly impact their willingness to engage with the platform. Based on these premises, the following hypothesis is proposed:

*H8*: Perceived risk influences adoption intention significantly.

Perceived disease threat (PDT) refers to individuals’ subjective perception of their susceptibility to a specific disease or their likelihood of becoming ill compared to others (Zheng et al., 2022). College students often face diverse health risks, such as stress-induced anxiety, irregular sleep patterns, and poor dietary habits. Individuals who perceive themselves as especially vulnerable to these issues may be more inclined to seek health information through short videos. Their heightened awareness of personal health threats can motivate them to adopt preventive behavior and engage more actively with health-related content. Based on these concepts, we propose the following hypothesis:

*H9*: Perceived disease threat influences adoption intention significantly.

Facilitating conditions (FC) refer to the perceived technical and equipment support that individuals believe they have when using a new system (Al-Adwan, 2020). Most college campuses offer reliable internet access, and students generally own smartphones, enabling seamless access to short video platforms. These facilitating conditions, including stable connectivity and familiar devices, alleviate usage barriers and promote sustained engagement with health-related content. Therefore, this study proposes:

*H10*: Facilitating conditions positively influence adoption behavior.

Adoption intention (AI) refers to individuals’ readiness to adopt and utilize information (Jeyaraj et al., 2023). Among college students, behavioral outcomes, such as liking, sharing, bookmarking, or applying health advice in real life reflect deeper engagement with the content. Students who demonstrate strong adoption intentions are more inclined to translate these intentions into actual behavior, particularly when the content resonates with their daily experiences and concerns. Drawing on this discourse, the following hypotheses are formulated:

*H11*: Adoption intention positively influences adoption behavior.

### 2.4 Construction of the conceptual model

**Figure 1:**
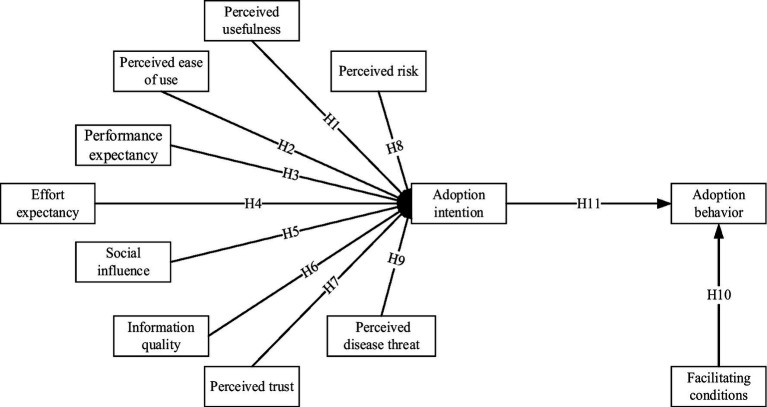
Model construction.

This study integrates the Technology Acceptance Model (TAM) and the Unified Theory of Acceptance and Use of Technology (UTAUT), incorporating eight variables: effort expectancy, performance expectancy, perceived usefulness, facilitating conditions, perceived ease of use, information quality, perceived trust, and social influence. These variables are used to construct a model that examines the factors influencing college students’ adoption of health information through short videos. The aim is to analyse how college student adopts health information via short videos, elucidate the factors influencing their adoption behavior, and explore the process of health information adoption in greater depth. The research hypotheses are illustrated in Figure 1.

## 3 Research method

### 3.1 Sample selection and data collection

Based on the hypotheses outlined above, this study initially employed in-depth interviews with 7 college students who have viewed health short videos to extract factors influencing their adoption behavior of health information through short videos. This approach aimed to refine the design of the survey questionnaire. This study has been approved by the Ethics Committee of Nanjing Medical University, Nanjing (Approval No. 2024781). Participants are informed about the purpose of the study and assured of anonymity before completing the questionnaire. After providing their consent to participate, they receive a survey questionnaire. Convenience sampling is adopted to efficiently collect a large number of responses while minimizing time and cost in data collection (Sinclair-Maragh, 2017). To mitigate common method bias (CMB), this study reduced the order effect by changing the order of the questions and measuring the predictor and criterion variables at different time points (Podsakoff et al., 2003).

The questionnaire designed for investigating factors influencing college students’ adoption of health information through short videos is divided into three sections, corresponding to the model’s independent, mediating, and dependent variables. The first part assesses ten independent variables: “information quality,” “perceived usefulness,” “perceived ease of use,” “effort expectancy,” “performance expectancy,” “social influence,” “perceived trust,” “perceived risk,” “perceived disease threat,” and “facilitating conditions.” The second part evaluates the mediating variable “adoption intention,” while the third part measures the dependent variable “adoption behavior.” The survey was administered using the Wenjuanxing platform, a widely used online survey tool in China, enabling efficient and cost-effective data collection with prompt feedback.

A total of 300 questionnaires were distributed in this survey, all targeting college students. Among them, 296 valid responses were received, resulting in an effective response rate of 98.7%. Table 1 provides an overview of the statistical information gathered from the collected questionnaires. According to existing research, the sample size for structural equation modeling should be determined based on the number of parameters within the model. The minimum sample size required for this study is 200, thus the sample size included in this study is adequate (Min et al., 2024).

**Table 1:** Demographic profile.

Measure	Items	Frequency	Percent
Age	18–23	207	69
	23–26	69	23
	Above 26	23	8
Gender	Male	146	49
	Female	150	51
Degrees	Below junior	15	5
	Undergraduate	189	64
	Master	70	24
	Above doctor	22	7
Major	Medical	19	6
	Non-medical	277	94
The frequency of users watching health short videos	Daily	82	27
	Weekly	127	43
	Monthly	88	30
The time of users watching health short videos daily	Less than 5 min	177	60
	5–10 min	93	31
	More than 10 min	26	9

### 3.2 Data analysis

“This study employed SPSS 22.0 (IBM Corp, Armonk, NY) to statistically analyse the 296 valid questionnaires collected, aiming to explore the factors influencing college students’ information adoption behavior related to health short videos. During the analysis, all tests were two-sided, with a significance level set at *α* = 0.05, and differences were considered statistically significant if the *p*-value was less than 0.05. The basic demographic characteristics of the study subjects, including gender, age, educational background, and major, were described using frequencies and proportions. The reliability of the questionnaire data was assessed using Cronbach’s Alpha coefficient (Wang et al., 2023), and the validity was evaluated using the Kaiser-Meyer-Olkin (KMO) (Lavebratt et al., 2014) and Bartlett’s test of sphericity (Dziuban and Shirkey, 1974) to assess the structural validity of the questionnaire.

## 4 Results

### 4.1 Common method bias

In this study, common method bias is procedurally and effectively controlled by adopting methods such as anonymous measurement and time-separated measurement. In addition, the collected data are checked for common method bias using Harman’s single factor test. The results of the analysis show that the overall variance of the first common factor is 48.68%, which is lower than the 50% criterion proposed by Podsakoff et al. (2003), so it can be concluded that there is no significant common method bias in this study.

### 4.2 Tests for multicollinearity

To examine whether multicollinearity exists among the variables, this study conducts a Variance Inflation Factor (VIF) test. The results are presented in Table 2. Since all VIF values are well below the critical threshold of 10, it can be concluded that multicollinearity is not a concern among the variables (Hair et al., 1995).

**Table 2:** Variance inflation factor test.

Construct	VIF
Perceived usefulness (PU)	3.773
Perceived ease of use (PEOU)	2.826
Performance expectancy (PE)	3.668
Effort expectancy (EE)	4.432
Social influence (SI)	4.411
Facilitating conditions (FC)	3.572
Information quality (IQ)	3.345
Perceived trust (PT)	3.794
Perceived risk (PR)	2.644
Perceived disease threat (PDT)	3.583

### 4.3 Reliability and validity

The reliability and validity of the model and its constructs were evaluated, with results detailed in Tables 3–5. The internal consistency, indicated by Cronbach’s alpha, ranged from 0.745 to 0.796, all exceeding the acceptable threshold of 0.7 (McNeish, 2018), demonstrating good internal consistency. Factor loadings for individual items were between 0.67 and 0.78, surpassing the threshold of 0.6 (Shevlin and Miles, 1998), indicating good convergent validity. The composite reliability (CR) for each construct ranged from 0.74 to 0.80, all above the threshold of 0.7 (Bacon et al., 1995), suggesting excellent structural reliability. The average variance extracted (AVE) for most constructs exceeded 0.5, except for performance expectancy, which had an AVE of 0.49. This minor deviation did not significantly impact the overall convergent validity of the measurement model (Wei et al., 2016). Additionally, the square roots of the AVEs for all variables were greater than their correlations with other variables, indicating good discriminant validity. Furthermore, the KMO test value for the scale used in this study was 0.980, well above the threshold of 0.70. The approximate chi-square was 6763.274 with a significance level of 0.000, less than 0.05, indicating strong construct validity and suitability for factor analysis.

**Table 3:** Model reliability and validity analysis.

Construct	Item	Item loading	Cronbach’s Alpha	Composite reliability (CR)	Average variance extracted (AVE)
Perceived usefulness (PU)	PU1	0.75	0.777	0.78	0.54
	PU2	0.75			
	PU3	0.71			
Perceived ease of use (PEOU)	PEOU1	0.72	0.771	0.77	0.53
	PEOU2	0.75			
	PEOU3	0.72			
Performance expectancy (PE)	PE1	0.69	0.745	0.74	0.49
	PE2	0.70			
	PE3	0.71			
Effort expectancy (EE)	EE1	0.74	0.781	0.78	0.54
	EE2	0.75			
	EE3	0.72			
Social influence (SI)	SI1	0.74	0.769	0.77	0.53
	SI2	0.73			
	SI3	0.71			
Facilitating conditions (FC)	FC1	0.73	0.757	0.78	0.54
	FC2	0.69			
	FC3	0.78			
Information quality (IQ)	IQ1	0.72	0.759	0.76	0.51
	IQ2	0.73			
	IQ3	0.70			
Perceived trust (PT)	PT1	0.77	0.788	0.79	0.56
	PT2	0.70			
	PT2	0.77			
Perceived risk (PR)	PR1	0.73	0.771	0.77	0.53
	PR2	0.75			
	PR3	0.71			
Perceived disease threat (PDT)	PDT1	0.76	0.796	0.80	0.57
	PDT2	0.74			
	PDT3	0.76			
Adoption intention (AI)	AI1	0.72	0.754	0.76	0.51
	AI2	0.72			
	AI3	0.70			
Adoption behavior (AB)	AB1	0.75	0.761	0.76	0.51
	AB2	0.73			
	AB3	0.67			

**Table 4:** Square AVE values and correlation coefficients of each variable.

Factors	PT	PR	PDT	AI	AB	IQ	FC	SI	EE	PE	PEOU	PU
PT	0.74											
PR	0.51	0.73										
PDT	0.57	0.51	0.70									
AI	0.49	0.42	0.48	0.74								
AB	0.49	0.43	0.49	0.41	0.73							
IQ	0.51	0.43	0.51	0.43	0.42	0.74						
FC	0.51	0.44	0.50	0.45	0.42	0.46	0.71					
SI	0.54	0.47	0.52	0.44	0.44	0.47	0.47	0.75				
EE	0.53	0.44	0.50	0.46	0.43	0.46	0.49	0.49	0.73			
PE	0.49	0.42	0.47	0.43	0.40	0.42	0.47	0.47	0.45	0.76		
PEOU	0.49	0.48	0.45	0.40	0.39	0.42	0.44	0.47	0.45	0.41	0.71	
PU	0.52	0.44	0.49	0.44	0.44	0.48	0.49	0.50	0.49	0.46	0.45	0.71

**Table 5:** KMO and Bartlett’s tests.

Measure		Value
KMO measure of sampling adequacy		0.980
Bartlett’s test of sphericity	Chi-square test	6763.274
	Degrees of freedom	630
	Statistical significance	0.000

### 4.4 Gap analysis

To examine the effects of gender, age, education, and major on each variable in the model, this study employed independent samples *t*-tests and one-way analysis of variance (ANOVA) to assess the impact of individual variables on others. The purpose of these analyses was to determine if these demographic factors significantly influenced the variables in the study. Independent samples *t*-tests were applied to gender and major, which have two groups of data each, while one-way ANOVA was used for age and education, as these categories involve more than two groups. Both independent samples *t*-tests and ANOVA rely on the significance value (Sig) to assess the significance of differences between variables: a Sig value less than 0.05 was considered statistically significant, indicating a meaningful difference between groups, whereas a value greater than 0.05 suggested no significant difference (Lakens, 2021).

#### 4.4.1 Gender’s influence on each variable

According to the results presented in Table 6, the Sig values for gender across various variables are all greater than 0.05. This finding suggests that gender does not significantly influence factors such as perceived usefulness in the model of health information adoption through health short videos among college students.

**Table 6:** Gender analysis of independent samples *t*-test for each variable.

Factors	Metrics	Levene’s test for equality of variances		Equality of means *t*-test		
		*F*	Statistical significance	*t*-value	Degrees of freedom	Sig.
PU	Assuming homoscedasticity	0.992	0.320	0.290	294	0.772
	Assuming heteroscedasticity			0.290	292.150	0.772
PEU	Assuming homoscedasticity	0.042	0.838	−0.223	294	0.824
	Assuming heteroscedasticity			−0.223	292.768	0.824
PE	Assuming homoscedasticity	2.639	0.105	0.545	294	0.586
	Assuming heteroscedasticity			0.547	285.599	0.585
EE	Assuming heteroscedasticity	0.241	0.624	0.935	294	0.351
	Assuming heteroscedasticity			0.936	293.785	0.350
SI	Assuming homoscedasticity	0.073	0.787	0.381	294	0.704
	Assuming heteroscedasticity			0.381	293.802	0.704
FC	Assuming homoscedasticity	0.464	0.496	0.991	294	0.323
	Assuming heteroscedasticity			0.992	291.400	0.322
IQ	Assuming homoscedasticity	0.739	0.391	0.821	294	0.412
	Assuming heteroscedasticity			0.823	286.953	0.411
PT	Assuming homoscedasticity	0.244	0.622	0.928	294	0.354
	Assuming heteroscedasticity			0.930	292.275	0.353
PR	Assuming homoscedasticity	0.651	0.420	0.347	294	0.729
	Assuming heteroscedasticity			0.348	289.697	0.728
PDT	Assuming homoscedasticity	1.911	0.168	1.001	294	0.317
	Assuming heteroscedasticity			1.003	290.254	0.317
AI	Assuming homoscedasticity	1.364	0.244	1.678	294	0.094
	Assuming heteroscedasticity			1.682	288.255	0.094
AB	Assuming homoscedasticity	0.794	0.373	0.506	294	0.613
	Assuming heteroscedasticity			0.507	291.852	0.613

#### 4.4.2 Major’s influence on each variable

According to the data in Table 7, the Sig value for Perceived Ease of Use is 0.048, which is less than 0.05. This indicates that different majors (medical and non-medical) significantly impact Perceived Ease of Use. However, these differences do not significantly affect Perceived Usefulness, Performance Expectancy, Effort Expectancy, or Facilitating Conditions.

**Table 7:** Major analysis of independent samples *t*-test for each variable.

Factors	Metrics	Levene’s test for equality of variances		Equality of means *t*-test		
		*F*	Statistical significance	*t*-value	Degrees of freedom	Sig.
PU	Assuming homoscedasticity	0.309	0.579	−0.227	294.000	0.821
	Assuming heteroscedasticity			−0.312	23.512	0.757
PEU	Assuming homoscedasticity	3.746	0.054	−1.984	294.000	0.048
	Assuming heteroscedasticity			−3.971	33.527	0.000
PE	Assuming homoscedasticity	0.379	0.539	−0.274	294.000	0.784
	Assuming heteroscedasticity			−0.384	23.780	0.704
EE	Assuming homoscedasticity	2.188	0.140	−0.475	294.000	0.635
	Assuming heteroscedasticity			−1.004	36.503	0.322
SI	Assuming homoscedasticity	2.632	0.106	−0.978	294.000	0.329
	Assuming heteroscedasticity			−1.954	33.426	0.059
FC	Assuming homoscedasticity	0.349	0.555	−0.766	294.000	0.444
	Assuming heteroscedasticity			−1.120	24.424	0.274
IQ	Assuming homoscedasticity	2.225	0.137	−0.696	294.000	0.487
	Assuming heteroscedasticity			−1.083	25.566	0.289
PT	Assuming homoscedasticity	0.854	0.356	−0.697	294.000	0.486
	Assuming heteroscedasticity			−1.093	25.721	0.284
PR	Assuming homoscedasticity	1.467	0.227	−1.341	294.000	0.181
	Assuming heteroscedasticity			−1.630	22.056	0.117
PDT	Assuming homoscedasticity	1.090	0.297	−0.218	294.000	0.827
	Assuming heteroscedasticity			−0.313	24.138	0.757
AI	Assuming homoscedasticity	0.653	0.420	−1.099	294.000	0.273
	Assuming heteroscedasticity			−1.552	23.881	0.134
AB	Assuming homoscedasticity	3.328	0.069	−1.007	294.000	0.315

#### 4.4.3 Age’s influence on each variable

According to the results presented in Table 8, the Sig values for age across all variables are greater than 0.05. This suggests that age does not have a significant impact on factors such as perceived usefulness in the model of health information adoption among college students using health short videos.

**Table 8:** Effect of age on model variables - analysis of variance.

Factors	Metrics	ANOVA				
		Square sum	Degrees of freedom	Mean square	*F*	Statistical significance
PU	Between-subjects design	1.092	2	0.546	0.936	0.393
	Randomized block design	170.912	293	0.583		
	Total	172.005	295			
PEU	Between-subjects design	0.98	2	0.49	0.722	0.487
	Randomized block design	198.923	293	0.679		
	Total	199.902	295			
PE	Between-subjects design	1.424	2	0.712	1.178	0.309
	Randomized block design	177.002	293	0.604		
	Total	178.426	295			
EE	Between-subjects design	2.935	2	1.468	2.45	0.088
	Randomized block design	175.519	293	0.599		
	Total	178.455	295			
SI	Between-subjects design	0.914	2	0.457	0.72	0.488
	Randomized block design	186.073	293	0.635		
	Total	186.988	295			
FC	Between-subjects design	2.929	2	1.464	2.425	0.09
	Randomized block design	176.951	293	0.604		
	Total	179.88	295			
IQ	Between-subjects design	1.613	2	0.806	1.409	0.246
	Randomized block design	167.69	293	0.572		
	Total	169.303	295			
PT	Between-subjects design	3.784	2	1.892	2.922	0.055
	Randomized block design	189.753	293	0.648		
	Total	193.538	295			
PR	Between-subjects design	2.192	2	1.096	1.545	0.215
	Randomized block design	207.873	293	0.709		
	Total	210.066	295			
PT	Between-subjects design	3.017	2	1.509	2.12	0.122
	Randomized block design	208.541	293	0.712		
	Total	211.558	295			
AI	Between-subjects design	0.182	2	0.091	0.151	0.86
	Randomized block design	176.849	293	0.604		
	Total	177.032	295			
AB	Between-subjects design	3.353	2	1.676	2.724	0.067
	Randomized block design	180.297	293	0.615		
	Total	183.65	295			

#### 4.4.4 Degrees influence on each variable

According to the data in Table 9, the Sig values for convenience conditions, information quality, and perceived disease threat are all less than 0.05. This indicates that different educational backgrounds significantly influence these factors. However, educational background does not significantly impact perceived usefulness, performance expectancy, perceived ease of use, effort expectancy, or perceived trust.

**Table 9:** Influence of degrees on variables - one-way analysis of variance.

Factors	Metrics	ANOVA				
		Square sum	Degrees of freedom	Mean square	*F*	Statistical significance
PE	Between-subjects design	2.730	3	0.910	1.569	0.197
	Randomized block design	169.275	292	0.580		
	Total	172.005	295			
PEU	Between-subjects design	0.443	3	0.148	0.216	0.885
	Randomized block design	199.459	292	0.683		
	Total	199.902	295			
PE	Between-subjects design	4.172	3	1.391	2.331	0.074
	Randomized block design	174.254	292	0.597		
	Total	178.426	295			
EE	Between-subjects design	2.197	3	0.732	1.213	0.305
	Randomized block design	176.258	292	0.604		
	Total	178.455	295			
SI	Between-subjects design	4.153	3	1.384	2.211	0.087
	Randomized block design	182.834	292	0.626		
	Total	186.988	295			
FC	Between-subjects design	5.943	3	1.981	3.326	0.020
	Randomized block design	173.937	292	0.596		
	Total	179.880	295			
IQ	Between-subjects design	5.036	3	1.679	2.984	0.032
	Randomized block design	164.267	292	0.563		
	Total	169.303	295			
PT	Between-subjects design	2.083	3	0.694	1.059	0.367
	Randomized block design	191.454	292	0.656		
	Total	193.538	295			
PR	Between-subjects design	2.029	3	0.676	0.950	0.417
	Randomized block design	208.036	292	0.712		
	Total	210.066	295			
PDT	Between-subjects design	7.269	3	2.423	3.463	0.017
	Randomized block design	204.289	292	0.700		
	Total	211.558	295			
AI	Between-subjects design	0.778	3	0.259	0.430	0.732
	Randomized block design	176.253	292	0.604		
	Total	177.032	295			
AB	Between-subjects design	3.891	3	1.297	2.107	0.099
	Randomized block design	179.759	292	0.616		
	Total	183.650	295			

### 4.5 Structural equation model

**Figure 2:**
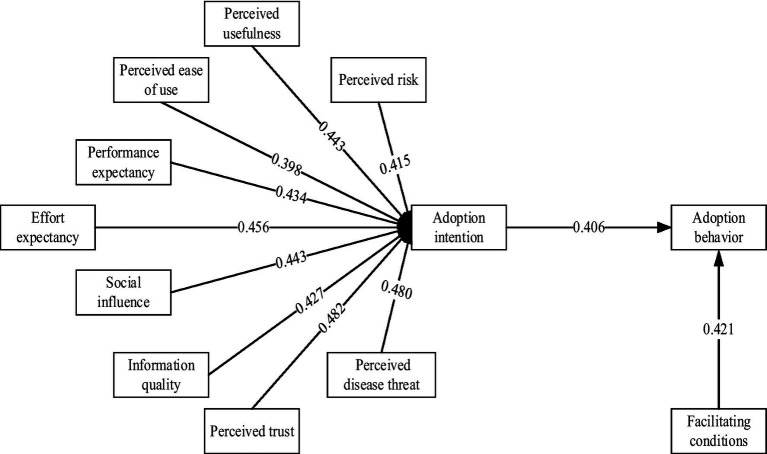
Structural equation model.

Structural Equation Modeling (SEM) is a statistical technique that constructs, estimates, and tests causal relationships between variables, making it suitable for analyzing variables that cannot be precisely measured. SEM requires a sample size of more than 200 cases; in this study, 296 valid responses were collected, enabling the use of SEM for analysis. The model of factors influencing college students’ adoption of health information from short videos is illustrated in Figure 2.

The evaluation of fit indices for the structural equation model primarily relies on the indicators in Table 10. Based on the data in Table 10, with *X*^2^/df = 1.103, GFI = 0.907, NFI = 0.918, CFI = 0.992, IFI = 0.992, PGFI = 0.719, PCFI = 0.831, PNFI = 0.769, RMSEA = 0.019, SRMR = 0.027, RFI = 0.902, TLI = 0.990, and AGFI = 0.924, it shows that the model has high goodness of fit.

**Table 10:** Analysis of overall model fit.

Hypothesis test	Value range of goodness of fit	Model goodness of fit	Result
*X*^2^/df	1–5	1.103	Supported
GFI	>0.9	0.907	Supported
NFI	>0.9	0.918	Supported
CFI	>0.9	0.992	Supported
IFI	>0.9	0.992	Supported
PGFI	>0.5	0.719	Supported
PCFI	>0.5	0.831	Supported
PNFI	>0.5	0.769	Supported
RMSEA	<0.08	0.019	Supported
SRMR	<0.08	0.027	Supported
RFI	>0.9	0.902	Supported
TLI	>0.9	0.990	Supported
AGFI	>0.9	0.924	Supported

In the structural equation model, standardized path coefficients indicate the extent to which independent variables influence dependent variables. Analysis of the path coefficients and *p*-values, as presented in Table 11, reveals that perceived usefulness, perceived ease of use, performance expectancy, effort expectancy, social influence, information quality, perceived trust, perceived risk, and perceived disease threat significantly impact the adoption intention path (*p* < 0.001). Furthermore, facilitating conditions have a significant impact on the adoption behavior path, and adoption intention significantly influences the adoption behavior path (*p* < 0.001).

**Table 11:** Results of path analysis and hypothesis testing.

Hypothesis	Path coefficient	*p*	Result
PU → AI	0.443	***	Supported
PEU → AI	0.398	***	Supported
PE → AI	0.434	***	Supported
EE → AI	0.456	***	Supported
SI → AI	0.443	***	Supported
IQ → AI	0.427	***	Supported
PT → AI	0.482	***	Supported
PR → AI	0.415	***	Supported
PDT → AI	0.480	***	Supported
AI → AB	0.406	***	Supported
FC → AB	0.421	***	Supported

*p < 0.05; **p < 0.01; ***p < 0.001.

This study employs the Gaussian Copula approach proposed by Park and Gupta (2012) to test for endogeneity. As shown in Table 12, the results of the endogeneity analysis indicate that the *p*-values for all paths are greater than 0.05. Thus, it can be inferred that there is no significant endogeneity issue in the structural model, and the obtained results are reliable (Hult et al., 2018).

**Table 12:** Gaussian copula approach.

Hypothesis	*p*-value
(GC) PU → AI	0.571
(GC) PEU → AI	0.834
(GC) PE → AI	0.867
(GC) EE → AI	0.726
(GC) SI → AI	0.238
(GC) IQ → AI	0.076
(GC) PT → AI	0.188
(GC) PR → AI	0.191
(GC) PDT → AI	0.926
(GC) AI → AB	0.572
(GC) FC → AB	0.541

## 5 Discussion

The study integrated the Technology Acceptance Model (TAM) and the Unified Theory of Acceptance and Use of Technology (UTAUT) to construct a comprehensive model of factors influencing college students’ adoption behavior of health short videos. The findings reveal that perceived usefulness, perceived ease of use, performance expectancy, effort expectancy, and social influence significantly and positively influence students’ adoption intention. Additionally, higher information quality and perceived trust enhance the willingness to adopt, while perceived risk and perceived disease threat can deter adoption behavior. Ultimately, facilitating conditions and adoption intention play crucial roles in promoting actual adoption behavior. These insights offer a theoretical foundation for optimizing the content and dissemination strategies of health short videos, which can improve health literacy among college students, foster healthy behavior, and guide the optimization of content and services on health video platforms to better address students’ health information needs.

### 5.1 Perceived ease of use and perceived usefulness significantly affected college students’ health information adoption

Consistent with previous research (Luo et al., 2024), this study demonstrates that PU and PEU positively and significantly influence college students’ intention to adopt health information. PU reflects college students’ belief in the practical effectiveness of health short videos for acquiring health knowledge, improving health behavior, and enhancing health awareness. When college students believe that watching health short videos empowers them to effortlessly master scientific dietary combinations or fitness regimens, and that this knowledge can exert a tangible positive impact on their daily lives, they are more inclined to deem health short videos as valuable. PEU primarily manifests in the platform’s operational convenience, the clarity and comprehensibility of content presentation, and the overall convenience of viewing. Platforms featuring user-friendly interfaces and robust search functionality, when combined with short video content that presents health knowledge in an engaging and entertaining manner, can significantly boost college students’ acceptance of and willingness to use such content. These findings align with in-depth interview results, indicating that students prefer platforms with high usability, such as Douyin and Little Red Book, due to their low learning curve and efficient access to valuable health information. They also prefer platforms that meet most of their information needs. Health short videos on these platforms provide rich content that addresses their health requirements, offering targeted solutions and advice that positively influence their health behavior and decisions. In the context of health information dissemination, health short video platforms should further optimize operational workflows and minimize users’ learning costs to ensure students can navigate effortlessly and access relevant information promptly. Content creators are encouraged to employ plain language and vivid case studies to transform complex health knowledge into approachable content, thereby lowering comprehension hurdles. These efforts can effectively reduce barriers to students’ understanding and adoption of health information.

### 5.2 Information quality and perceived trust positively significantly affect the information adoption of health short video college students

The results of this study indicate that IQ and PT significantly and positively influence college students’ adoption of health short videos. This finding aligns with Pang et al., who demonstrated that IQ and PT exert positive impacts on the intention to adopt health information (Deng et al., 2015). High-quality information content is typically marked by elevated accuracy, comprehensiveness, and authority, thereby boosting college students’ trust in and acceptance of the health knowledge delivered via health short videos. When college students perceive the health information in short videos as legitimate, reliable, and verifiable, particularly when it is sourced from government agencies or reputable international organizations, they are more likely to regard the information as trustworthy and adopt it. Perceived trust is primarily shaped by the creator’s professional background, the authority of the content, and positive user reviews and interactive feedback within the platform. These elements jointly inform users’ judgments of the content’s authenticity and credibility. Therefore, to enhance students’ trust and promote adoption, platforms should institute rigorous content review mechanisms and encourage contributions from certified medical professionals. Content creators should prioritize citing authoritative information sources, emphasize their professional credentials, and foster interactive user engagement through comments and feedback, thereby elevating both the credibility and reach of health short videos. Perceived risk and perceived disease threat significantly affect the information adoption of health short video college students.

Previous studies have demonstrated that PR can have a negative impact on an individual’s behavioral intentions (Li, 2025). This study also confirms this process. When college students encounter health short videos, when college students encounter health short videos, concerns about inaccurate or misleading information and its potential irreversible effects on their health can elevate their perceived risk, thereby reducing their willingness to adopt such content. The increase in perceived risk not only suppresses information adoption behavior but may also trigger negative emotions such as anxiety and fear, leading students to avoid healthy content and further diminishing their receptiveness. In contrast, PDT exhibits a positive influence. Faced with academic and personal stress, college students often tend to neglect health issues in their daily lives. However, physical symptoms or external health events may heighten their risk awareness and prompt greater motivation to seek health information. This risk-driven awareness can strengthen their intention to adopt health short video content. Based on the above findings, the design of health short video content should seek to balance perceived risk. On one hand, creators ought to explicitly indicate information sources, target audiences, and risk warnings in videos, while avoiding exaggerated or alarmist language to mitigate unwarranted concern. On the other hand, platforms can implement content labeling mechanisms such as “reassuring labels” or “doctor recommendations” to enhance user trust and alleviate uncertainty.

### 5.3 Facilitating conditions positively significantly affected the information adoption of health short video college students

In the structural equation model analysis, the path coefficient for FC is 0.421, indicating a significant positive effect on health information adoption, consistent with the conclusions of previous studies (Dwivedi et al., 2019). FC refers to the technical and equipment support available to college students when using short video platforms, enabling greater access to and engagement with health-related content. Specifically, the ease of operation on short video platforms, low-cost access to health information, abundant resources of health short videos, efficient personalized recommendation mechanisms, mature resource retrieval systems, and well-organized content sections (as exemplified by Little Red Book, which segments into categories such as “fitness and shaping,” “scientific popularization,” “psychology,” “weight loss,” among others, rather than a standalone “health popularization” section) collectively enhance the convenience of accessing health information for college student. Therefore, these conditions not only enhance the user experience on short video platforms but also significantly increase their willingness to adopt health information from these platforms. Therefore, short video platforms should continue to optimize the user experience by developing more intelligent personalized recommendation systems, efficient content classification mechanisms, and robust retrieval functions, thereby enhancing the convenience and relevance of health information access. Moreover, universities and public health institutions can collaborate to establish campus-specific health video channels tailored to college students’ needs. Such initiatives may include simplifying interfaces, providing technical support to reduce usage barriers, and ultimately expanding the coverage and dissemination impact of health information.

### 5.4 Performance expectancy and effort expectancy positively and significantly affect the information adoption of health short video college students

The results indicate that PE and EE significantly influence college students’ information adoption. This finding is consistent with previous research findings (Al-Sharafi et al., 2023), emphasizing that when short video platforms provide high-quality health information and a convenient user experience, students are more likely to engage with and adopt the information presented. PE involves students’ expectations of obtaining high-quality, authoritative health information tailored to their needs through browsing health short videos, aimed at enhancing health knowledge and addressing practical health issues. For instance, students might use short video platforms before physical education classes to search for effective warm-up exercises, precautions, and sports techniques, or when facing health concerns like weight loss or fitness, they may seek effective methods or fitness guidance through short videos. EE refers to the convenience with which students can access the desired health information on short video platforms. If a platform allows users to easily access the information they need, students are more likely to continue using it. Due to their user-friendly interface and low time cost, short video platforms encourage students to prefer this method of obtaining health information, further increasing their willingness and actual behavior to adopt health information. In summary, platforms and content creators should collaborate to improve content quality and user experience by producing concise, targeted, and information-dense videos. These efforts should enable users to access practical and relevant health advice with minimal effort, thereby fostering deeper engagement and promoting the adoption of health behavior.

### 5.5 Social influence positively significantly affects the information adoption of health short video college students

The findings support the hypothesis and are consistent with previous studies that SI has a considerable positive impact on user intention to adopt information (Hameed et al., 2024; Gonzalez-Tamayo et al., 2024). This suggests that advice and recommendations from others have a significant impact on shaping college students’ intention to adopt health short videos. Most college students indicated that they began to pay attention to and use health short videos for information due to the influence of friends and family. As part of the short video user base, college students are particularly susceptible to the opinions of others. During decision-making processes, due to uncertainty about outcomes, advice from family, friends, and doctors, as well as recommendations from teachers and classmates, often significantly influences their decisions on adopting information. Particularly in the realm of health information, college students are inclined to adopt health short videos recommended by family members, peers, and authoritative sources to improve their health conditions. Therefore, it is essential to promote a socially driven approach to health information dissemination. Platforms can deploy social recommendation functionalities such as “Friends Are Viewing” and “Family Recommendations” to enhance interpersonal trust and facilitate information sharing among users. Moreover, health education programs can be integrated with peer support mechanisms, leveraging online check-ins and experience-sharing mechanisms to trigger positive imitation effects and foster the interactive diffusion of health information within college student communities.

## 6 Implications

### 6.1 Implications for theory

This study integrates the TAM model with the UTAUT model to explore the factors affecting the continuous adoption of health short videos among Chinese college students. It investigates how content quality and presentation styles of health short videos influence students’ perceived value, emotional responses, and information adoption intention. By integrating these two theoretical models, the study enriches and extends the existing theoretical framework, unraveling the complex psychological mechanisms underlying students’ adoption behavior and identifying key driving factors. The findings indicate that perceived usefulness, perceived ease of use, performance expectancy, effort expectancy, social influence, information quality, and perceived trust have a significant positive impact on adoption intention. In contrast, perceived risk and perceived disease threat exert a negative influence. Facilitating conditions positively affect adoption behavior, and adoption intention significantly influences adoption behavior. By refining the content and presentation characteristics of health short videos, this study provides new perspectives for the field of health science communication and offers important guidance for the development and optimization of health science communication products.

### 6.2 Implications for practice

Given that university students are among the most active users of short video platforms, health-focused platforms must address their specific needs. To accommodate their fast-paced lifestyles and preference for mobile-first interactions, platforms should streamline access to health services and optimize interfaces for mobile devices. Integrating functions such as online appointments, teleconsultations, and health screenings into a single platform can provide a convenient and efficient “one-stop” service tailored to students’ needs. Furthermore, enhancing interactive and social features such as comment sections, peer support groups, and shareable health tips can significantly increase user engagement by tapping into students’ preference for peer-driven information and real-time interaction. Recommendation algorithms should be refined to exclude commercial or low-quality content and prioritize evidence-based health information, thereby meeting students’ needs for reliable and accessible resources.

For university students, ensuring the credibility of health-related short videos is essential, as they often depend on digital sources for information but may lack the critical literacy to distinguish accurate content from misinformation. Given the widespread appeal of infotainment among young audiences, platforms should implement strict content moderation policies to curb the spread of misinformation and commercial bias. Establishing expert-reviewed content channels, partnering with university health centers, and incorporating user reporting mechanisms can enhance platform credibility and mitigate perceived risks. In addition, personalized recommendation systems should account for the diverse health concerns of university students, including mental health, sleep disorders, nutrition, and physical fitness. By analyzing user behavior and preferences, platforms can provide personalized, age-appropriate health content that improves comprehension and strengthens relevance and engagement. To balance diversity and simplicity in recommendations, platforms should ensure broad content coverage while accommodating individual learning styles and health needs.

## 7 Limitations

While this study has yielded valuable insights into the impact of health short videos on information adoption behavior among Chinese college students, several limitations remain. Firstly, the sample size of 296, although adequate for structural equation model analysis, is relatively small and may limit the generalizability and extrapolation of the findings. Secondly, the study primarily focuses on college students, which may not fully represent the health information adoption behavior of people from different age groups, professions, and educational backgrounds. In addition, the convenience sampling method used in this study lacks randomization, which may lead to sample bias, and the independent variables used are conventional variables, limiting to some extent, the study’s wider contribution to the literature. Future research could enhance representativeness by expanding the sample to include individuals from various regions, cultures, and socioeconomic backgrounds or by adopting more scientific sampling methods. Additionally, while the study design employs structural equation model to analyse the path relationships between variables, it is limited by its cross-sectional nature and cannot capture the dynamic processes and causal relationships over time. Future research could further explore the development of theoretical models specifically tailored to the dissemination of health short videos to more accurately identify influencing factors and dissemination mechanisms. These approaches could provide more comprehensive and in-depth insights for optimizing health short video content and improving health communication strategies.

## Data Availability

The raw data supporting the conclusions of this article will be made available by the authors, without undue reservation.
